# Comparative Study of Swab Culture and Tissue Biopsy in Burn Wound Sepsis: A Tertiary Centre Experience

**DOI:** 10.7759/cureus.99588

**Published:** 2025-12-18

**Authors:** Praveen Gopi, Sulfekar M.S, Shenol Sasankan

**Affiliations:** 1 Department of Urology, Mersey and West Lancashire Teaching Hospitals NHS Trust, Prescot, GBR; 2 Department of General Surgery, Government Medical College, Thrissur, IND; 3 Department of General Surgery, Government Medical College, Thiruvananthapuram, IND; 4 Department of Plastic surgery, Cosmetiq Clinic, Thiruvananthapuram, IND

**Keywords:** bacterial count, burn, burn unit, sepsis, swab culture

## Abstract

Infection remains a critical cause of mortality among patients with burn injuries, with sepsis and subsequent multiorgan failure being the predominant reasons for death in intensive burn care units. The emergence of nosocomial pathogens in this population makes early identification of infection essential for predicting septicemia and reducing mortality rates. This study aimed to compare the efficacy of surface swab culture versus tissue biopsy culture in identifying pathogenic organisms in burn wounds and examine the correlation between bacterial counts and clinical outcomes.​ A prospective study was conducted enrolling 50 burn patients admitted to the burns ward at Thrissur Medical College, Kerala, India. For each patient, surface swabs and tissue biopsies were collected and processed using conventional culture methods. The statistical analysis was performed using chi-square tests.

There was a significant relationship between total body surface area (TBSA) affected and clinical outcome, with a mean TBSA of 45.8%. Notably, 42/50 (84%; p < 0.05) of patients sustained third-degree burns, which was statistically significant in its impact on outcome. Bacterial growth was observed in 48 (96%) of surface swab cultures and 49 (98%) of tissue biopsy samples, with a concordance rate of 19/50 (38%). The most frequently isolated organisms were Pseudomonas aeruginosa 17(34%), Staphylococcus aureus, Klebsiella, and Acinetobacter. A bacterial load greater than 10^5 CFU/ml on quantitative biopsy was significantly associated with poor outcome, as 18/19 (94.7%) patients who died demonstrated counts above this threshold (p < 0.05). In conclusion, both wound surface swab and tissue biopsy cultures were effective in demonstrating pathogen presence, but tissue biopsy was superior in the context of deeper wound invasion. Incorporating tissue biopsy culture into routine management algorithms for high‑risk burn patients may therefore improve prognostication, optimize antibiotic stewardship, and ultimately reduce sepsis‑related deaths in burn units.

## Introduction

Skin is one of the largest organs in the human body, with a surface area of 1.5 to 2.0 m² and a weight of 6 kg to 10 kg in an average adult. An intact human skin surface is vital for the preservation of body fluid homeostasis, thermoregulation, and protection against infection. The skin also has immunological, neurosensory, and metabolic functions, including vitamin D metabolism [1]. As an essential component of the nonspecific immune system, the skin protects the host from potential environmental pathogens. Breaches in this protective barrier constitute a form of immunocompromise that predisposes patients to infection.

Burns are considered one of the most common and severe forms of trauma. The incidence of burn injuries is high in developing countries, creating a significant public health problem. Major demographic factors associated with a high risk of burns include high population density, illiteracy, and poverty [2]. Most cases are caused by electrical burns, fire, hot liquids, and contact with hot surfaces. Burn wound infections are among the most significant and potentially serious complications occurring in the acute period following injury [3]. It is estimated that 50% to 75% of mortality in burn patients after initial resuscitation is attributable to infection [4]. The rate of nosocomial infection is higher in burn patients due to factors such as the nature of the burn injury, its depth and extent, the patient's immunocompromised status, invasive diagnostic and therapeutic procedures, and prolonged hospital stays. Since the skin barrier is disrupted, preventing infections becomes a challenge, and the heavily colonized burn unit environment with resistant microorganisms can predispose patients to cross-contamination [5].

The primary reservoirs for microorganisms that colonize the burns of new patients are the burn wound surfaces and the gastrointestinal tracts of patients [6]. Microorganisms can be transmitted by the hands of healthcare workers, fomites, hydrotherapy water, and, in some reports, by air [7]. The flora of the burn wound also influences the infection risk and invasive potential [8]. Early contaminants of the wound surface after burning are most likely skin flora, particularly gram-positive organisms [9]. Over time, gram-negative organisms become more prevalent, but gram-positive organisms remain the predominant flora. Following colonization, these organisms may invade viable tissue depending on their invasive capacity, local wound factors, and the degree of patient immunosuppression. Disseminated infections occur when sub-eschar tissue is invaded [10].

There is a direct correlation between the degree of bacterial wound contamination and the risk of wound sepsis. The most commonly used techniques for microbial monitoring of burn wounds are swab culture and biopsy culture. Surface cultures are useful for identifying organisms present on the burn wound and the predominant burn wound flora; however, these samples are insufficient to differentiate between burn wound colonization and burn wound infection [11]. It is now accepted that quantitative cultures of biopsy samples, histopathological examination, and clinical assessment are required to distinguish colonization from actual infection. Most studies have used a bacterial level of 1 × 10⁵ bacteria per gram of tissue as the diagnostic criterion for invasive infection and septicemia [12].

In our institution, the major cause of mortality in burn patients is wound sepsis. For the diagnosis of wound infection, we routinely perform surface swab cultures to assess microbiological status after initiating empirical antibiotic coverage. However, the results can be inconclusive when a deeper infection occurs. As a result, proper assessment of microbiological status is lacking in the later stages of wound infection, increasing the risk of sepsis. Therefore, an alternative investigative method is required to analyze the infective pathology. In this study, a prospective analysis was performed to determine the bacteriological profile and to evaluate the possibility of predicting septicemia in burn patients through the use of both wound surface and tissue culture techniques.

## Materials and methods

This prospective observational study was conducted in the burns ward of the Government Medical College, Thrissur, Kerala, India, between August 2012 and August 2013. A total of 50 consecutive patients admitted with partial or full thickness burns affecting over 10% of total body surface area (TBSA), as assessed by Wallace's rule, were enrolled in the study. The enrolled patients provided written informed consent. Patients younger than 13 years and those with burns involving less than 10% of TBSA were excluded, as pediatric and minor burns are not managed in our unit. Approval was obtained from the Institutional Ethics Committee of Medical College, Thrissur, and all patient details were anonymized to maintain confidentiality.​

Microbiological assessment included collection of both surface swab samples and full‑thickness biopsy specimens from each patient for culture analysis, either within one week of admission or at the time of clinical wound sepsis. Clinical follow-up continued until discharge or in-hospital mortality, ensuring comprehensive data capture for outcome analysis during the one-year study period.

Procedure

Wound swab and biopsy specimens were collected simultaneously from the wound of each patient. Samples were procured from the leading edge of the wound sites showing signs of infection, such as discoloration, bad odor, and rapid separation of the eschar or presence of pus. To obtain a surface swab, dressings and topical agents were removed with gauze soaked in sterile saline. An area measuring 4 cm^2^ was swabbed using two sterile swab sticks. For dry wounds, the swab was moistened with sterile saline before swabbing.

Tissue biopsies were obtained after cleaning the burn wound surface with sterile saline. Two parallel incisions were made in the skin, approximately 1 cm to 2 cm in length and 1.5 cm apart. Sterile tissue forceps were used to elevate the tissue, and using a sterile scalpel, a sample was obtained from the subcutaneous tissue at sufficient depth with a small portion of healthy underlying fat as described by Loebl et al. [13]. The biopsy specimen was placed within sterile saline-moistened gauze in a leakproof sterile container.

Analysis of burn wound specimens

Both samples were transported immediately to the laboratory, processed, and analyzed to identify all potential pathogens and to perform antibiotic susceptibility testing. A Gram stain was done. Qualitative analyses of the samples were obtained with inoculation of blood, MacConkey agar, and chocolate agar. Quantitative tissue cultures were obtained from tissue biopsy samples, and colony counts were performed after 24 hours of inoculation to obtain the bacterial count per ml of tissue. No growth up to 48 hours was considered negative. Initially, the cross-section is made by sterile forceps and a scalpel, followed by the making of an impression smear. The smear is stained and placed over the Neubauer counting chamber, and a bacterial count is performed.

Statistical analysis

Data collected were thoroughly checked and entered into an MS Excel (Microsoft Corp., Redmond, WA, USA) spreadsheet. All statistical analyses were performed using SPSS Statistics software version 21 (IBM Corp., Armonk, NY, USA) with a p-value < 0.05 accepted as statistically significant. Univariate analysis was used to compare the relationship between age, gender, burn depth, comorbidity, total surface burn area, hospital duration, and burn etiology. Positive results of quantitative biopsy versus surface swab cultures, and the chi‑square test was used for categorical variables.

## Results

This prospective comparative study analyzed 50 burn patients over one year, of which 31 (62%) survived, and 19 (38%) died. Patient demographics showed a female predominance (32 patients (64%) female and 18 (36%) male). Notably, among the deceased patients, there were 13 (41.6%) females and 6 (33.3%) males, though this difference was not statistically significant (p=0.610). Age of patients ranged from 16 to 78 years, with the largest cohort (17 patients; 34%) in the 21 to 30 years age group and a mean age of 39.24 years (SD ± 16.28), but there was no significant correlation between age and patient outcome (Table 1). Regarding comorbidities, the majority of patients (38; 76%) had none, whilst 12 patients (24%) had comorbid conditions including diabetes (n=5), hypertension (n=3), coronary artery disease (n=2), acute liver disease (n=1), and chronic kidney disease (n=1) (Table 2).

**Table 1:** Demographic summary of burn patients

Variable	Category	Frequency	Percentage
Outcome	Survived	31	62.0
	Expired	19	38.0
Age Group (yrs)	21-30	17	34.0
	31-40	10	20.0
	41-50	7	14.0
	51-60	7	14.0
	61-70	4	8.0
	71-80	2	4.0
Sex	Male	18	36.0
	Female	32	64.0

**Table 2:** Burn characteristics and comorbidities TBSA: Total body surface area

Variable	Category	Frequency	Percentage
Depth of burn	2nd degree	8	16.0
	3rd degree	42	84.0
TBSA (%)	20-30	9	18.0
	31-40	11	22.0
	41-50	9	18.0
	51-60	15	30.0
	61-70	6	12.0
Comorbidity	Diabetes	5	10.0
	Hypertension	3	6.0
	Coronary disease	2	4.0
	None	38	76.0

The TBSA affected by burns ranged from 20% to 70% with a mean of 45.83% (SD ± 13.91). The distribution of TBSA involvement revealed that 15 patients (30%) sustained 51% to 60% burns, 11 (22%) sustained 31% to 40%, nine patients each (18%) sustained 20% to 30% and 41% to 50% burns, respectively, and six patients (12%) sustained 61% to 70% burns. Significantly, 24 of 31 survivors (77%) had burns affecting less than 50% TBSA, whereas 12 of 19 deceased patients (63%) had burns exceeding 50% TBSA, a statistically significant difference (p=0.003).

Burn depth revealed that the majority of patients (42; 84%) sustained third-degree burns, whilst only eight (16%) had second-degree burns (Table 2). Notably, all patients with second-degree burns survived (100%), whereas the mortality rate among those with third-degree burns was 45.2%, representing a statistically significant difference (p=0.016). Regarding etiology, 29 patients (58%) sustained flame burns and 21 patients (42%) sustained scald burns. The cause of burns was predominantly accidental (44 patients; 88%), with only six patients (12%) sustaining suicidal burns. Of the accidental burns, 25 were scald-related, and 19 were flame-related.

Hospital stay duration had a mean of 20.18 days (SD ± 10.87), ranging from a minimum of six days to a maximum of 60 days. The majority of patients (16; 32%) were hospitalized for 11 to 20 days, followed by 14 patients (28%) who stayed 21 to 30 days. Mean hospital duration was 21.42 ± 9.67 days amongst survivors and 18.16 ± 12.59 days amongst deceased patients, which was not statistically significant (p=0.308).

Microbiological analysis revealed bacterial growth in 48 of 50 (96%) surface swab samples and 49 of 50 (98%) tissue culture samples. Positive tissue biopsies demonstrated a single bacterial species in 41 cases and polymicrobial infection (two species) in nine cases, whilst surface swab cultures showed single species in 26 cases and polymicrobial communities in 22 cases, with only 26 of 50 (52%) surface swabs yielding conclusive results. *Pseudomonas aeruginosa* was the predominant pathogen in both culture types, isolated alone or in combination with other pathogens in 31 tissue biopsies (62%) compared to 17 surface swab samples (34%) (Table 3). Additional pathogens identified in tissue cultures included *Acinetobacter Baumannii* (20%), *Staphylococcus Aureus* (14%), *Escherichia coli* (12%), and *Klebsiella Pneumoniae* (8%), whilst swab cultures identified *S. Aureus* (8%), *A. Baumanni*i (6%), and *E. coli *(4%). Culture concordance between tissue biopsy and surface swab for positive bacterial growth was achieved in only 19 of 50 patients (38%), with *P. aeruginosa* demonstrating the highest concordance rate at 12 of 19 (63%) concordant cases. 

**Table 3:** Microbiological findings (swab and tissue cultures)

Organism	Swab frequency (%)	Tissue frequency (%)
Pseudomonas aeruginosa	17 (34.0)	22 (44.0)
Staphylococcus Aureus	4 (8.0)	5 (10.0)
Acinetobacter Baumannii	3 (6.0)	7 (14.0)
Escherichia coli	2 (4.0)	5 (10.0)
Klebsiella pneumoniae	--	1 (2.0)
Mixed	22 (44.0)	9 (18.0)
Sterile	2 (4.0)	1 (2.0)

Among the studied cases, 19 patients (38.0%) had a bacterial count of ≤100,000 CFU/ml, while 31 patients (62.0%) showed counts exceeding 100,000 CFU/ml. Of those with bacterial counts ≤100,000 CFU/ml, 18 patients (58.1%) survived, and nine (5.3%) expired. In contrast, among patients with bacterial counts >100,000 CFU/ml, 13 (41.9%) survived, whereas 18 (94.7%) expired, indicating a markedly higher mortality rate associated with higher bacterial counts, which was statistically significant (p < 0.001).

## Discussion

Burn wound infection is one of the most frequent and serious complications in patients with burn injuries. This prospective study included 50 patients admitted to the burn ward of Government Medical College, Thrissur (Kerala, IND). The objective was to compare the efficacy of surface swab and tissue biopsy cultures in detecting pathogens and predicting sepsis using bacterial counts.​

All patients were followed until discharge or death. Of the 50 patients included, 19 (38%) died, reflecting a considerable mortality rate. A similar finding was documented by Sjoberg et al. in Zimbabwe [14]. The mean age of patients was 39.24 years, with most (34%) in the 21- to 30-year age group. No statistically significant difference was observed in the mean age of the deceased and surviving patients. Neelam et al. showed a similar conclusion with no significant relation between age and outcome of the patient [15]. In contrast, Ahamed et al. from Pakistan found that advanced age was a significant risk factor for burn mortality [16]. In the present study, the incidence was higher in females (32, 64%) compared to males (18, 32%), although the outcome did not differ by sex. Contrasting trends exist, with Alaghehbandan et al. [17] and Ibnouzahir et al. [18] reporting a greater burn incidence among males, while Cutilas et al. and Liu et al. [19,20] found a predominance in females. Local factors, such as involvement in firework industries, may explain male vulnerability. Around 24% of patients had comorbidities, including diabetes, hypertension, and coronary artery disease.​

Flame burns constituted the predominant etiology in this cohort, accounting for 29 cases (58%), whereas scald injuries were responsible for 21 cases (42%), indicating that flame‑related mechanisms were a substantially more common cause of burn injury than hot liquid exposure in the study population. Males were disproportionately affected by scalds, whereas females more commonly suffered flame burns. Both flames and scalds showed higher incidence among females (72% and 52%, respectively). Accidental burns were primarily due to flames (52%), while all suicidal burns in the cohort were among females, via flame. The mean percentage of TBSA burned was 45.8%, with most cases in the 51% to 60% range. Alaghehbandan et al. and Ying et al. [17,21] described a high incidence of scald among females. In contrast, Panjeshahin et al. and Barret et al. [22,23] reported that males sustain flame burns more frequently than females. The TBSA correlated strongly with outcome; 77% of survivors had <50% TBSA involvement, while 63% of deaths occurred in those with >50% TBSA. Depth was also significant; 42 (84%) sustained third-degree burns, and 16% (eight) second-degree burns. All patients with second-degree burns survived, whereas the mortality rate for third-degree burns was 45%. The mean hospital stay was 20.18 days, with no statistically significant impact on outcome.​

Bacterial isolates were found in 48 (96%) of surface swab samples and 49 (98%) of tissue biopsies. Tissue biopsies demonstrated slightly higher sensitivity for pathogen detection compared to swabs. However, 44% of swab results were inconclusive due to polymicrobial growth or inability to perform antibiotic sensitivity testing, likely reflecting specimen collection from colonized tissue. Tissue biopsy resolved this limitation, accurately identifying pathogens and allowing effective targeted therapy. *Pseudomonas aeruginosa* was the most frequently isolated pathogen in both cultures, followed by *Staphylococcus* in swab samples and* Acinetobacter* in biopsies.* Klebsiella* and *Pseudomonas *predominated during the second week post-burn, while *S. aureus *was more common during the initial five to seven days. McManus [24] also reported* P. aeruginosa* as the most common microorganism in burn wounds. Meanwhile,* S. aureus* is reported as the most frequent in most of the studies [15]. Moist, devitalized burn tissue provides an ideal environment for colonization, particularly by* P. aeruginosa*, exacerbated by immunocompromised status [25]. *Pseudomonas* was detected in 17 (34%) swabs and 31 (62%) biopsies. *Pseudomonas* was present in 58% of survivors and 68% of deceased patients, supporting its association with invasive wound infection and sepsis.​

The concordance rate between swab and biopsy cultures for positive bacterial growth was 38%, with *Pseudomonas* achieving 63% concordance. Previous studies reported varying levels of correlation. Bill et al. [26] found 79% concordance, Steer et al. [27] 54%, and Basak et al. [28] 72%. Concordance was highest for *S. aureus *and *A. baumannii* in some series. Biopsy samples are methodologically preferable for quantifying organism load, which is critical for management decisions. In this study, a significant bacterial count (>10^5 CFU/ml) was found in 27 (54%) samples, and in 18/19 (94.7%) of deaths versus 13/31 (41.9%) survivors, a statistically significant finding. Steer et al. [25] found a positive correlation between total bacterial count (by biopsy) and white cell count, while Herruzo-Cabrera et al. [29] demonstrated the utility of semiquantitative swab methods with biopsy at the threshold of 105 organisms per ml. These results support using a threshold of >10^5 CFU/ml to predict sepsis in burns. Significant associations were observed between biopsy-positive cultures and TBSA, burn depth, and bacterial count, enabling improved prognosis prediction and sepsis risk stratification in burn patients.

Limitations

This study has several important limitations that warrant consideration. The research was conducted at a single institution over a one-year period with a relatively small sample size of 50 patients, which may limit the generalizability of findings to other populations and burn care settings. The study excluded patients under 13 years of age and those with less than 10% TBSA involvement, thereby restricting the applicability of results to more severe burns in adult populations. Additionally, conventional culture methods were employed for microbiological analysis, which may not capture fastidious or slow-growing organisms. The study design was observational in nature without a control group, and the retrospective categorization of patients into survivor and deceased groups, combined with a relatively high mortality rate (38%), raises questions about the applicability of findings to centres with different outcomes or treatment protocols. Furthermore, the low concordance rate (38%) between swab and tissue cultures suggests that both methods have diagnostic limitations, and a larger multicentre study would be beneficial to validate these findings across diverse clinical settings and patient populations.

## Conclusions

Wound infection is the leading cause of mortality in burn patients. The early identification and treatment of the wound infection is of prime importance in reducing the mortality rate. The outcome for the patient is dependent on different factors, like the TBSA, depth of burn, positive biopsy culture, and significant bacterial count. By linking high TBSA, third‑degree burns, and elevated quantitative biopsy counts with mortality, the findings support using tissue biopsy selectively in high‑risk patients to improve risk stratification and timing of intervention. Integrating biopsy‑based microbiology into burn care pathways can therefore help guide targeted antimicrobial therapy, limit unnecessary broad‑spectrum antibiotic use, and potentially reduce sepsis‑related deaths in intensive burn care units. 
